# Draft Genome Sequences of Two Strains of Bifidobacterium dentium Isolated from a Crude Fecal Extract Used for Fecal Microbiota Transplantation in the Republic of Korea

**DOI:** 10.1128/MRA.00197-20

**Published:** 2020-06-04

**Authors:** Erin Simpson, Fatemeh Sanjar, Dennis Wylie, Sowon Park, Hong Koh, Jaewoong Bae, Sun-Young Kook, Seokjin Kim, Hyun Jung Kim

**Affiliations:** a Department of Biomedical Engineering, The University of Texas at Austin, Austin, Texas, USA; b Center for Biomedical Research Support, The University of Texas at Austin, Austin, Texas, USA; c Department of Pediatrics, Yonsei University College of Medicine, Severance Fecal Microbiota Transplantation Center, Severance Hospital, Seoul, Republic of Korea; d Hecto Innovation Lab, Hecto Co., Ltd., Seoul, Republic of Korea; e R&D Institute, BioEleven Co., Ltd., Seoul, Republic of Korea; f Department of Oncology, Dell Medical School, The University of Texas at Austin, Austin, Texas, USA; University of Maryland School of Medicine

## Abstract

We present the draft genome sequences of two Bifidobacterium dentium strains isolated from a fecal extract for fecal microbiota transplantation at a hospital in the Republic of Korea. Phylogenetic and functional analyses were performed to understand the physiological characteristics and functions of *Bifidobacterium* spp. in the human intestine.

## ANNOUNCEMENT

*Bifidobacterium* is a genus of Gram-positive, anaerobic, nonmotile, and non-spore-forming bacteria considered to be key contributors to the healthy commensal gut microbiome (1). Predicted to constitute less than 10% of the adult human microbiota, *Bifidobacterium* has long been studied as an important class of probiotics due to many beneficial functions, including the inhibition of pathogen colonization, host immune modulation, and the maintenance of intestinal barrier function (2–5). These physiological functions are essential for gut homeostasis and the prevention of numerous gastrointestinal disorders (1, 6). To better understand the underlying mechanisms of these beneficial functions and how they can be utilized therapeutically, it is critical to identify the genetic composition of naturally occurring *Bifidobacterium* populations in healthy individuals (7).

In this report, we describe draft genome sequences of two Bifidobacterium dentium strains (FMTc1 and FMTc3) isolated from the processed fecal extract provided by a qualified, healthy South Korean donor. This crude fecal extract derived from a fecal sample has been used for fecal microbiota transplantation (FMT) for patients with inflammatory bowel disease in the Severance Fecal Microbiota Transplantation Center at Severance Hospital in the Republic of Korea (institutional review board [IRB] approval number 4-2017-0223). The fecal specimen was collected and processed according to the standard operating procedures at a commercial stool bank, BioEleven Co. in the Republic of Korea (IRB approval number P01-201803-31-009; ClinicalTrials registration number NCT03399188). Healthy fecal donors were screened based on the IRB-approved protocol. A fresh fecal sample collected from a fecal donor was resuspended (0.2% [wt/vol]) with a sterilized saline solution (0.9% [wt/vol]) containing autoclaved glycerol (final concentration, 12.5% [vol/vol]). The fecal homogenate was then filtered (cutoff size, 300 μm) and homogenized at room temperature under aerobic conditions for 10 min (BagMixer; Interscience). Aliquots were stored at −80°C until use.

The crude fecal extract obtained from BioEleven Co. was used to isolate the reported strains by performing single-colony isolation using *Bifidobacterium*-selective medium (BSM) agar (Anaerobe Systems) at 37°C in an anaerobic glove box (5% H_2_, 5% CO_2_, and 90% N_2_). After incubation for 36 h, individual colonies were independently subcultured in 5 ml of liquid BSM for 48 h at 37°C in the same anaerobic glove box, without shaking.

Genomic DNA was isolated and purified (QIAamp PowerFecal DNA kit; Qiagen), qualified (NanoDrop UV spectrophotometer; Thermo Fisher Scientific), and then submitted for whole-genome sequencing (CosmosID). Libraries were prepared using the Ion Xpress Plus fragment library kit (Thermo Fisher Scientific) according to the manufacturer’s protocol and then sequenced with the Ion S5 next-generation sequencing system (Thermo Fisher Scientific). A summary of genome assembly statistics and strain information is provided in Table 1. The raw single-end reads were trimmed and processed using BBDuk (BBMap v36.49) with a reading quality trimming parameter of 20 (8). The genome assembly was achieved using the SPAdes assembler (v3.9.0) (9). Completeness of the assembled isolates was evaluated using the CheckM lineage_wf function (v1.0.13) (10). Genome annotation of assembled isolates was achieved using the Prokka Annotation Pipeline (v1.13) with the added parameters mincontiglen 200 and compliant (11). Default parameters were used for all software unless otherwise specified.

**TABLE 1:** Genome assembly statistics and strain information

Strain	GenBank accession no.	Total no. of reads	Genome coverage (×)	Avg contig coverage (×)	No. of contigs	No. of genes	No. of coding sequences	Genome size (bp)	No. of tRNAs	G+C content (%)	*N*_50_ (bp)
FMTc1	ERS4331523	3,907,594	269.3952	54.1136	29	2,202	2,143	2,538,385	58	58.49	137,183
FMTc3	ERS4331524	3,921,209	270.2438	50.4725	33	2,209	2,152	2,539,231	56	58.49	162,381

Genome sizes for isolates FMTc1 and FMTc3 were 2,538,385 and 2,539,231 bp, respectively. Annotation of each genome revealed 2,202 genes, 2,143 protein-coding regions, and 58 tRNA genes in B. dentium FMTc1 and 2,209 genes, 2,152 protein-coding regions, and 56 tRNA genes in B. dentium FMTc3, with a G+C content of 58.49% in both isolates. Both isolates were found to produce toxins A and HipA and to possess ESX secretion systems.

### Data availability.

Draft genome sequences for B. dentium FMTc1 and B. dentium FMTc3 are available in GenBank under accession numbers ERS4331523 and ERS4331524, respectively. The accession numbers for the genome assemblies are JAAWWM000000000 (for B. dentium FMTc1) and JAAWWN000000000 (for B. dentium FMTc3), under BioProject numbers PRJNA622788 and PRJNA622792.
